# Supplementary material for: Viruses Infecting Greenhood Orchids (Pterostylidinae) in Eastern Australia
Source: Viruses. 2022 Feb 10;14(2):365. doi: 10.3390/v14020365 (PMC8876172; doi:10.3390/v14020365)
Supplement: Supplementary file 1 [file viruses-14-00365-s001.zip › Supplementary_Materials.pdf]

Table S1. Assembly parameters for SPAdes.

Sample	K-mers Used	Minimum Input Read Length
13365	21,33,55 / 21,33,55,89,93,97 *	100 bp
13392	21,33,55,77,89,93,97,107,117,127	130 bp
13394	21,33,55,77,89,93,97,107,117,127	130 bp
13395	21,33,55,77,89,93,97,107,117,127	130 bp
13396	21,33,55,77,89,93,97,107,117,127	130 bp
13399	21,33,55,77,89,93,97,107,117,127	100 bp
13402	21,33,55,77,89,93,97,107,117,127	130 bp
13421	21,33,55,77,89,93,97,107,117,127	130 bp
13430	21,33,55,77,89,93,97,107,117,127	130 bp
13438	21,33,55,77,89,93,97,107,117,127	130 bp
13442	21,33,55,77,89,93,97,107,117,127	130 bp
GF	21,33,55,77 / 21,33,55,77,89,93,97,107,117,127 *	100 bp
HR	21,33,55,77 / 21,33,55,77,89,93,97,107,117,127 *	100 bp
PI-1	21,33,55,77 / 21,33,55,77,89,93,97,107,117,127 *	100 bp
PI-2	21,33,55,77 / 21,33,55,77,89,93,97,107,117,127 *	100 bp

* Assembly was done twice, and any incomplete virus contigs from the first run were used as trusted contigs for the second run.

Table S2. Gene-specific primers used in rapid amplification of cDNA ends (RACE) and reverse transcription (RT)-PCR.

Primer Sequence (5' to 3')	Target Isolate	Usage
TATGACCTAGAAGAAATGCTCAGAC	PtBV-13365, L Segment	5'-RACE First PCR
CCAGCATTGAGAGCACATGGATAC	PtBV-13365, L Segment	5'-RACE Nested PCR
TGTCTTAGTGAATCTTGCATGTATAG	PtBV-13365, L Segment	5'-RACE Sequencing
TGAAGATGACAAAATTGTCAGGAGTGG	PtBV-13365, L Segment	3'-RACE First PCR
AATGAAGTTTCTTCCTCACTAGC	PtBV-13365, L Segment	3'-RACE Nested PCR
TTTCTAATAATCCTTCAGTTTTGC	PtBV-13365, L Segment	3'-RACE Sequencing
CTGTCTTCTATTTTATTTGGATCCAC	PtBV-13365, M Segment	5'-RACE First PCR
TGCAGTTGGGCAAACCCATAC	PtBV-13365, M Segment	5'-RACE Nested PCR
ATATTTGTTTGCCATTGTCC	PtBV-13365, M Segment	5'-RACE Sequencing
TCTCCACTACTCAAAACAACCTGA-TAAGG	PtBV-13365, M Segment	3'-RACE First PCR
TTCTGAAATAGAACAATCACCTG	PtBV-13365, M Segment	3'-RACE Nested PCR
CCTTTCAAAACACATTGCTC	PtBV-13365, M Segment	3'-RACE Sequencing
AAGAATATCTGAAGATGA-TAATGGTTCATATCC	PtBV-13365, S Segment	5'-RACE First PCR
TTTAATTGGAGAACCAGATATATCC	PtBV-13365, S Segment	5'-RACE Nested PCR
ATACATGGCAATGAAATAAATTG	PtBV-13365, S Segment	5'-RACE Sequencing
CATTGTTCAAAAATGTTGTTACAG	PtBV-13365, S Segment	3'-RACE First PCR
TAGTGCAAAGCTTTCAGATTCCAAACC	PtBV-13365, S Segment	3'-RACE Nested PCR
TTTCTTTCTATTTCTGATGATAGAGC	PtBV-13365, S Segment	3'-RACE Sequencing
TTGTGGAAAAGTTAACTCGCAC	PtPV	5'-RACE First PCR
GAGGCAAACCACCCAGTCAG	PtPV	5'-RACE Nested PCR
CAAGGAAAGGGAAGGATCTC	PtPV	5'-RACE Sequencing
AAGCCGCGTACGTATTGATCC	PtPV	3'-RACE First PCR
CCAGTTTATCTGGTGGTAGCTTGAG	PtPV	3'-RACE Nested PCR
TTCTTTGAGAACCAAGAATGAG	PtPV	3'-RACE Sequencing
GTGTATTCCATGCATCTGGC	PtAEV	5'-RACE cDNA Synthesis (Coding Strand)
TAACGCATTAACTGGTTGCC	PtAEV	5'-RACE First PCR (Coding Strand)
ATTGCGCATCGTGTCATAAG	PtAEV	5'-RACE Nested PCR (Coding Strand)
TACTCACCATGTGGACTGAC	PtAEV	5'-RACE Sequencing (Coding Strand)
AAAGCAAAACAGTGAGTGGC	PtAEV	5'-RACE cDNA Synthesis (Complemen-tary Strand)
GTTTCTTGTGTTGGGTGACG	PtAEV	5'-RACE First PCR (Complementary Strand)
AAGGTTCGAGGTGACTAACG	PtAEV	5'-RACE Nested PCR (Complementary Strand)
GATTGAACGGGTTGGAATGG	PtAEV	5'-RACE Sequencing (Complementary Strand)
AGTCAGCGATATTGCGCTCC	PtAV-13365	5'-RACE cDNA Synthesis (Coding Strand)
GCGGTGTACTTGTCACCGAC	PtAV-13365	5'-RACE First PCR (Coding Strand)
CTCAACCAGATCCTTCGGTTCG	PtAV-13365	5'-RACE Nested PCR (Coding Strand)
AGATAACGGAAGACCTTCTG	PtAV-13365	5'-RACE Sequencing (Coding Strand)

AGATGGAATCGTAGCATACCCAATG	PtAV-13365	5'-RACE cDNA Synthesis (Complementary Strand)
CTCTACGGTGACTACTCTTTATTCACG	PtAV-13365	5'-RACE First PCR (Complementary Strand)
TGATATACTTGCGTGGTGTG	PtAV-13365	5'-RACE Nested PCR (Complementary Strand)
TCTGAGGACAGCATCGCAGC	PtAV-13365	5'-RACE Sequencing (Complementary Strand)
ATCTCGTCAAAGTCGGGC	PtAV-13438-2	5'-RACE cDNA Synthesis (Coding Strand)
CCTTTCCTCCTTGACAGTTG	PtAV-13438-2	5'-RACE First PCR (Coding Strand)
AGATGAAGGTTGTGACGGTC	PtAV-13438-2	5'-RACE Nested PCR (Coding Strand)
TCATAGGTGATCCCTTGAGC	PtAV-13438-2	5'-RACE Sequencing (Coding Strand)
CACGGGAATCACCATTTCTG	PtAV-13438-2	5'-RACE cDNA Synthesis (Complementary Strand)
GCAGGCAATAAGGATCTTTCG	PtAV-13438-2	5'-RACE First PCR (Complementary Strand)
AGTTTGGGAACGACATCATC	PtAV-13438-2	5'-RACE Nested PCR (Complementary Strand)
CTTTTGACCTCCTGGGAGAC	PtAV-13438-2	5'-RACE Sequencing (Complementary Strand)
AGGGTAATTAGCCGCATCACG	PtTV-13365	5'-RACE cDNA Synthesis (Coding Strand)
CCTCATGAACACAAATCGCATCATG	PtTV-13365	5'-RACE First PCR (Coding Strand)
AGTGTTGCTGACAGTACATC	PtTV-13365	5'-RACE Nested PCR (Coding Strand)
GGCAGCAGTCATCGGCTG	PtTV-13365	5'-RACE Sequencing (Coding Strand)
TTTCGCACAAACGTTGACATCTG	PtTV-13365	5'-RACE cDNA Synthesis (Complementary Strand)
TTGTATACTTGGAACCATCGGACC	PtTV-13365	5'-RACE First PCR (Complementary Strand)
GACTGGTCAATTAAACGAGTG	PtTV-13365	5'-RACE Nested PCR (Complementary Strand)
GTGGAGAAGGCTCGGTTTGC	PtTV-13365	5'-RACE Sequencing (Complementary Strand)
GCACCGGATCAATCAACC	PtTV-13421-2	5'-RACE cDNA Synthesis (Coding Strand)
TGAAACAGAATCGCAGCATG	PtTV-13421-2	5'-RACE First PCR (Coding Strand)
GTACTTGCCTGTTCAAACCG	PtTV-13421-2	5'-RACE Nested PCR (Coding Strand)
GAACAGTGAGTATGCCAGAG	PtTV-13421-2	5'-RACE Sequencing (Coding Strand)
CACGGGAATCACCATTTCTG	PtTV-13421-2	5'-RACE cDNA Synthesis (Complementary Strand)
GCAGGCAATAAGGATCTTTCG	PtTV-13421-2	5'-RACE First PCR (Complementary Strand)
AGTTTGGGAACGACATCATC	PtTV-13421-2	5'-RACE Nested PCR (Complementary Strand)
CTTTTGACCTCCTGGGAGAC	PtTV-13421-2	5'-RACE Sequencing (Complementary Strand)

AGCAGTGAATACAAAGTCTTTGG	PtBV Nucleocapsid Gene	RT-PCR (653 bp Amplicon) Forward Primer and Sequencing
GAGGCACAATTTTCACTTCAAC	PtBV Nucleocapsid Gene	RT-PCR (653 bp Amplicon) Reverse Primer and Sequencing
TAAACAGTTGGTTGAGCAGC	PtAEV Glycotransferase Domain	RT-PCR (305 bp Amplicon) Forward Prime and Sequencing
GACAAGTATGTGCACAGTGG	PtAEV Glycotransferase Domain	RT-PCR (305 bp Amplicon) Reverse Primer and Sequencing
GGTATGATACCTAGTGGTTCTCT	PtAV1 ORF2	RT-PCR (432 bp Amplicon) Forward Prime and Sequencing
CGCTAAAAACTTTGGCTTACG	PtAV1 ORF2	RT-PCR (432 bp Amplicon) Reverse Primer and Sequencing
CTATATCACATGCGCTCTCCG	PtAV1 ORF2 Amplicon of PtAV-13395-1	Sequencing (Forward)
TAAGATCTTGCTTGTGCCCTG	PtAV1 ORF2 Amplicon of PtAV-13395-1	Sequencing (Reverse)
ATACATTACATGCGCATTACA	PtAV1 ORF2 Amplicon of PtAV-13395-2	Sequencing (Forward)
CAAAATCTTGCTCGTGCCCAA	PtAV1 ORF2 Amplicon of PtAV-13395-2	Sequencing (Reverse)
GGAGGTAATTCGAACGATGC	PtAV2 (PtAV-13438-2) ORF2	RT-PCR (496 bp Amplicon) Forward Primer and Sequencing
AGGGAATATCATGCCTCCTG	PtAV2 (PtAV-13438-2) ORF2	RT-PCR (496 bp Amplicon) Reverse Primer and Sequencing
CACGTACGCTCCATAGTG	PtPV Capsid Gene	RT-PCR (646 bp Amplicon) Forward Primer and Sequencing
CCCTGGTTCCTCGTCTAC	PtPV Capsid Gene	RT-PCR (646 bp Amplicon) Reverse Primer and Sequencing
GATGGACTACGGAGCAGAAC	PtTV (Excluding PtTV-13421-2) ORF2	RT-PCR (662 bp Amplicon) Forward Primer and Sequencing
ACATCATGAACGTGCCYACTATT	PtTV (Excluding PtTV-13421-2) ORF2	RT-PCR (662 bp Amplicon) Reverse Primer and Sequencing
GATGGACTATGGAGCCGATC	PtTV-13421-2 ORF2	RT-PCR (662 bp Amplicon) Forward Primer and Sequencing
ACATCAAAAATGTGCTCACAACC	PtTV-13421-2 ORF2	RT-PCR (662 bp Amplicon) Reverse Primer and Sequencing

Table S3. Sources of orthotospovirus and Dugbe virus sequences.

Genus	Virus	Abbreviation	GenBank Accession Number		
			L	M	S
Orthotospovirus	Alstroemeria necrotic streak virus	ANSV	MG696851	MG696852 (NSm) MK275259 (GP)	MG696853
Orthotospovirus	Alstroemeria yellow spot virus	AYSV	MF469033	MF469034	MF469035
Orthotospovirus	Barleria chlorosis-associated virus	BCaV	MW251496	MW251497	MW251498
Orthotospovirus	Bean necrotic mosaic virus	BeNMV	JF417980	JN587269	JN587268
Orthotospovirus	Calla lily chlorotic spot virus	CCSV	FJ822962	DQ256125	DQ256123
Orthotospovirus	Capsicum chlorosis virus	CaCV	DQ256124	FJ822961	AY867502
Orthotospovirus	Chilli yellow ringspot virus	CYRSV	MH779497	MH779496	MH779495
Orthotospovirus	Chrysanthemum stem necrosis virus	CSNV	KM114546	KM114547	KM114548
Orthotospovirus	Groundnut chlorotic fan-spot virus	GCFSV	KP146140	U42555	U27809
Orthotospovirus	Groundnut ringspot virus	GRSV	MH742956	KP146141	AF080526
Orthotospovirus	Groundnut bud necrosis virus	GBNV	AF025538	AB061773	AB038343
Orthotospovirus	Hippeastrum chlorotic spot virus	HCRV	HG763861	MH742957	MH742958
Orthotospovirus	Impatiens necrotic spot virus	INSV	X93218	JX833565	JX833564
Orthotospovirus	Iris yellow spot virus	IYSV	FJ623474	M74904	X66972
Orthotospovirus	Melon severe mosaic virus	MSMV	KX698424	AF214014	AF001387
Orthotospovirus	Melon yellow spot virus	MYSV	AB061774	KX698423	KX698422
Orthotospovirus	Mulberry vein banding virus	MVBaV	KM819698	KM819699	KM819701
Orthotospovirus	Pepper chlorotic spot virus	PCSV	KX247379	KX247378	KX247377
Orthotospovirus	Polygonum ringspot virus	PolRSV	KJ541746	KJ541745	KJ541744
Orthotospovirus	Scadoxus chlorotic ringspot virus	ScCRV	MW080808	MW080809	MW080810
Orthotospovirus	Soybean vein necrosis virus	SVNV	HQ728385	HQ728386	HQ728387
Orthotospovirus	Tomato chlorotic spot virus	TCSV	MH742959	MH742960	MH742961
Orthotospovirus	Tomato necrotic ringspot virus	TNRV	MW256413	MW256414	MW256415
Orthotospovirus	Tomato necrotic spot associated virus	TNSaV	KT984754	KT984753	KM355773
Orthotospovirus	Tomato spotted wilt virus	TSWV	KT717691	KT717692	KT717693
Orthotospovirus	Tomato yellow ring virus	TYRV	JN560178	JN560177	AY686718
Orthotospovirus	Tomato zonate spot virus	TZSV	EF552435	EF552434	EF552433
Orthotospovirus	Watermelon bud necrosis virus	WBNV	GU735408	GU584185	GU584184
Orthotospovirus	Watermelon silver mottle virus	WSMoV	AF133128	U75379	U78734
Orthotospovirus	Zucchini lethal chlorosis virus	ZLCV	KU641378	KU641379	KU641380
Orthonairovirus	Dugbe virus	DUGV	U15018		

NSm: non-structural protein, M segment; GP: glycoprotein precursor.

Table S4. Sources of endornavirus and grapevine leafroll-associated virus 1 sequences.

Genus	Virus	Abbreviation	GenBank Accession Number
Alphaendornavirus	Agaricus bisporus endornavirus 1	AbEV1	KY357509
Alphaendornavirus	Basella alba endornavirus 1	BaEV1	AB844264
Alphaendornavirus	Bell pepper endornavirus	BPEV	KX977569
Alphaendornavirus	Cluster bean endornavirus 1	CBEV1	MG764084
Alphaendornavirus	Cucumis melo endornavirus	CmEV	KT727022
Alphaendornavirus	Erysiphe cichoracearum endornavirus	EcEV	KT388110
Alphaendornavirus	Grapevine endophyte endornavirus	GEEV	JX678977
Alphaendornavirus	Helianthus annuus endornavirus	HaEV	MF362666
Alphaendornavirus	Helicobasidium mompa endornavirus 1	HmEV1	AB218287
Alphaendornavirus	Hordeum vulgare endornavirus	HvEV	KT721705
Alphaendornavirus	Hot pepper endornavirus	HPEV	KR080326
Alphaendornavirus	Lagenaria siceraria endornavirus	LsEV	KF562072
Alphaendornavirus	Oryza rufipogon endornavirus	OrEV	AB014344
Alphaendornavirus	Oryza sativa endornavirus	OsEV	D32136
Alphaendornavirus	Persea americana endornavirus 1	PaEV1	JN880414
Alphaendornavirus	Phaseolus vulgaris endornavirus 1	PvEV1	KT456287
Alphaendornavirus	Phaseolus vulgaris endornavirus 2	PvEV2	KT456288
Alphaendornavirus	Phaseolus vulgaris endornavirus 3	PvEV3	MG242064
Alphaendornavirus	Phytophthora endornavirus 1	PEV1	AJ877914
Alphaendornavirus	Rhizoctonia cerealis endornavirus 1	RcEV1	KF311065
Alphaendornavirus	Rhizoctonia solani endornavirus 2	RsEV2	KT823701
Alphaendornavirus	Vicia faba endornavirus	VfEV	AJ000929
Alphaendornavirus	Winged bean endornavirus 1	WBEV1	LC144945
Alphaendornavirus	Yerba mate endornavirus	YmEV	KJ634409
Betaendornavirus	Tuber aestivum endornavirus	TaEV	HQ380014
Ampelovirus	Grapevine leafroll-associated virus 1	GLRaV1	JQ023131

Table S5. Sources of amalgavirus, Zygosaccharomyces bailii virus Z and Amasya cherry disease associated chrysovirus sequences.

Genus	Virus	Abbreviation	GenBank Accession Number
Amalgavirus	Allium cepa amalgavirus 1	AcAV1	BK010347
Amalgavirus	Allium cepa amalgavirus 2	AcAV2	BK010348
Amalgavirus	Blueberry latent virus	BBLV	HM029246
Amalgavirus	Rhododendron virus A	RhVA	HQ128706
Amalgavirus	Southern tomato virus	STV	EF442780
Amalgavirus	Spinach amalgavirus 1	SpAV1	KY695011
Amalgavirus	Vicia cryptic virus M	VCVM	EU371896
Amalgavirus	Zoostera marina amalgavirus 1	ZmAV1	KY783316
Amalgavirus	Zoostera marina amalgavirus 2	ZmAV2	KY783317
Zybavirus	Zygosaccharomyces bailii virus Z	ZbV-Z	KU200450
Alphachrysovirus	Amasya cherry disease associated chrysovirus	ACDACV	AJ781166

Table S6. Sources of totivirus and Amasya cherry disease associated chrysovirus sequences.

Genus	Virus	Abbreviation	GenBank Accession Number
Totivirus	Pterostylis sanguinea totivirus A	PtSTVA	KU291927
Totivirus	Saccharomyces cerevisiae virus L-A	ScVLA	J04692
Totivirus	Saccharomyces cerevisiae virus LBCLa	ScVLBC	U01060
Totivirus	Scheffersomyces segobiensis virus L	SsV1	KC610514
Totivirus	Tuber aestivum virus 1	TaV1	HQ158596
Totivirus	Ustilago maydis virus H1	UmVH1	U01059
Totivirus	Xanthophyllomyces dendrorhous virus L1A	XdVL1A	JN997472
Totivirus	Xanthophyllomyces dendrorhous virus L1B	XdVL1B	JN997473
Alphachrysovirus	Amasya cherry disease associated chrysovirus	ACDACV	AJ781166

Table S7. Sources of polerovirus and enamovirus sequences.

Genus	Virus	Abbreviation	GenBank Accession Number
Polerovirus	Beet chlorosis virus	BChV	AF352024
Polerovirus	Beet mild yellowing virus	BMYV	X83110
Polerovirus	Beet western yellows virus	BWYV	AF473561
Polerovirus	Carrot red leaf virus	CtRLV	AY695933
Polerovirus	Cereal yellow dwarf virus RPS	CYDV-RPS	AF235168
Polerovirus	Cereal yellow dwarf virus RPV	CYDV-RPV	L25299
Polerovirus	Chickpea chlorotic stunt virus	CpCSV	AY956384
Polerovirus	Cotton leafroll dwarf virus	CLDV	GU167940
Polerovirus	Cucurbit aphid-borne yellows virus	CAbYV	X76931
Polerovirus	Faba bean polerovirus 1	FBPV1	MH464873
Polerovirus	Maize yellow dwarf virus RMV	MYDVRMV	KC921392
Polerovirus	Maize yellow mosaic virus	MaYMV	KU248489
Polerovirus	Melon aphid-borne yellows virus	MABYV	EU000534
Polerovirus	Pepo aphid-borne yellows virus	PABYV	KU315177
Polerovirus	Pepper vein yellows virus 1	PeVYV-1	AB594828
Polerovirus	Pepper vein yellows virus 2	PeVYV-2	HM439608
Polerovirus	Pepper vein yellows virus 3	PeVYV-3	KP326573
Polerovirus	Pepper vein yellows virus 4	PeVYV-4	KU999109
Polerovirus	Pepper vein yellows virus 5	PeVYV-5	KY523072
Polerovirus	Pepper vein yellows virus 6	PeVYV-6	LT559483
Polerovirus	Potato leafroll virus	PLRV	D00530
Polerovirus	Pumpkin polerovirus	PuPV	MG800833
Polerovirus	Suakwa aphid-borne yellows virus	SAbYV	JQ700308
Polerovirus	Sugarcane yellow leaf virus	ScYLV	AF157029
Polerovirus	Tobacco vein distorting virus	TVDV	EF529624
Polerovirus	Turnip yellows virus	TuYV	X13063
Enamovirus	Alfalfa enamovirus 1	AEV1	KU297983
Enamovirus	Citrus vein enation virus	CVEV	HF679486
Enamovirus	Grapevine enamovirus 1	GEV1	KX645875
Enamovirus	Pea enation mosaic virus 1	PEMV1	HM439775

Table S8. Numbers of paired reads before and after trimming.

Sample	Raw Data	Assembly*	Mapping**
13365***	27,654,877 27,099,376 28,022,954	19,020,569 17,616,117 22,763,158	22,338,523 22,056,390 24,106,815
13392	28,337,237	12,522,368	13,524,332
13394	24,458,259	10,070,330	11,569,876
13395	20,649,715	7,922,337	9,044,726
13396	24,316,593	11,199,377	12,221,386
13399	28,257,471	11,741,532	12,647,860
13402	20,913,028	8,931,944	N/A
13421	24,638,458	10,813,580	11,905,838
13430	28,686,506	11,388,182	12,791,672
13438	23,698,290	10,320,423	11,623,667
13442	21,592,081	8,567,868	9,754,992
GF	2,430,909	937,766	1,006,729
HR	3,010,297	1,037,906	1,134,158
PI-1	1,172,786	959,962	N/A
PI-2	3,344,246	2,718,254	N/A

* Trimmed to 100 bp or 130 bp, see Table S2. ** Trimmed to 30 bp. *** This sample was sequenced using three lanes of a flow cell and thus contains three datasets. N/A Not applicable as the process was not performed.

Table S9. Substitution models used in reconstructing maximum likelihood trees in Figures 3 to 7.

Figure	Nucleotide Substitution Models
3	RdRp: GTR+F+R5, GP: GTR+F+R5, NSm: GTR+F+G4, NSs: TPM3u+F+I+G4, N: GTR+F+I+G4
4	GTR+F+R5
5	TVM+F+R3
6	GTR+F+G4
7	Four conserved regions from 5' to 3': TIM2e+R4, SYM+I+G4, TIM2e+I+G4, TIM2e+R3

RdRp: RNA dependent RNA polymerase; GP: glycoprotein precursor; NSm: non-structural protein, M segment; NSs: non-structural protein, S segment; N: nucleocapsid protein.

Table S10. Closest matches of each Pterostylis blotch virus protein from BLASTP searches of the NCBI non-redundant protein sequence database.

Virus Protein	Isolate	Virus Name of the Closest Hit	GenBank Accession Number of the Closest Hit	E-value	Query Coverage (%)	Pairwise Identity (%)
RdRp	PtBV-13365	Barleria chlorosis-associated virus	QVY47424.1	0.0	99	50.8
	PtBV-GF	Barleria chlorosis-associated virus	QVY47424.1	0.0	99	50.9
	PtBV-HR	Barleria chlorosis-associated virus	QVY47424.1	0.0	99	50.7
	PtBV-13399	Barleria chlorosis-associated virus	QVY47419.1	0.0	99	50.7
NSm	PtBV-13365	groundnut ringspot virus	AAM47012.1	5.E-93	84	50.2
	PtBV-GF	groundnut ringspot virus	AAM47012.1	4.E-93	84	50.2
	PtBV-HR	groundnut ringspot virus	AAM47012.1	2.E-93	84	50.6
	PtBV-13399	groundnut ringspot virus	QBC75818.1	1.E-91	79	51.6
GP	PtBV-13365	Barleria chlorosis-associated virus	QVY47426.1	0.0	94	44.3
	PtBV-GF	Barleria chlorosis-associated virus	QVY47426.1	0.0	94	44.2
	PtBV-HR	Barleria chlorosis-associated virus	QVY47426.1	0.0	94	44.1
	PtBV-13399	Barleria chlorosis-associated virus	QVY47426.1	0.0	94	44.4
NSs	PtBV-13365	tomato chlorotic spot virus	QBC75815.1	2.E-15	93	21.1
	PtBV-GF	groundnut ringspot virus	AYA95028.1	2.E-16	96	23.7
	PtBV-HR	groundnut ringspot virus	AYA95028.1	2.E-16	96	23.7
	PtBV-13399	impatiens necrotic spot virus	QAU55706.1	1.E-12	93	23.1
N	PtBV-13365	bean necrotic mosaic virus	YP_006468900.1	6.E-45	96	39.7
	PtBV-GF	bean necrotic mosaic virus	YP_006468900.1	6.E-45	96	39.7
	PtBV-HR	bean necrotic mosaic virus	YP_006468900.1	6.E-45	96	39.7
	PtBV-13399	bean necrotic mosaic virus	YP_006468900.1	3.E-44	96	39.3

RdRp: RNA dependent RNA polymerase; GP: glycoprotein precursor; NSm: non-structural protein, M segment; NSs: non-structural protein, S segment; N: nucleocapsid protein.

Table S11. Pairwise sequence identities (%) of the orthotospoviruses identified in this study.

**Gene/Protein**		PtBV-13365	PtBV-GF	PtBV-HR	PtBV-13399
RdRp			99.5	99.4	97.8
NSm			99.7	98.2	97.9
GP	PtBV-13365		99.7	99.3	98.9
NSs			98.2	98.2	93.4
N			100.0	100.0	99.6
RdRp		98.10		99.7	97.8
NSm		99.18		98.5	97.6
GP	PtBV-GF	99.22		99.3	98.9
NSs		98.30		100.0	94.0
N		98.37		100.0	99.6
RdRp		98.2	99.1		97.7
NSm		98.3	98.5		96.6
GP	PtBV-HR	98.0	98.1		98.5
NSs		98.0	99.0		94.0
N		97.8	98.9		99.6
RdRp		90.1	90.2	90.2	
NSm		96.1	95.7	95.6	
GP	PtBV-13399	96.6	96.7	96.5	
NSs		91.4	91.7	91.4	
N		92.0	93.1	92.5	

Nucleic acid identities (in black font) are shown in the bottom left half of the table while amino acid identities (in red front) are shown in the top right half. RdRp: RNA dependent RNA polymerase; GP: glycoprotein precursor; NSm: non-structural protein, M segment; NSs: non-structural protein, S segment; N: nucleocapsid protein.

Table S12. Pairwise sequence identities (%) of the amalgaviruses identified in this study.

	PtAV-13365	PtAV-13392	PtAV-13394	PtAV-13395-1	PtAV-13395-2	PtAV-13396	PtAV-13421	PtAV-13430	PtAV-13438-1	PtAV-13438-2	PtAV-13442
PtAV-13365		88.7<br>90.3	89.1<br>90.1	89.2<br>90.2	96.5<br>96.5	96.8<br>96.8	99.3<br>99.2	88.7<br>90.2	99.5<br>99.5	40.6<br>46.7	96.9<br>96.9
PtAV-13392	85.1<br>86.5		92.5<br>93.1	92.6<br>93.2	88.9<br>90.5	88.9<br>90.5	89.0<br>90.8	99.5<br>99.5	88.9<br>90.5	41.0<br>46.8	88.9<br>90.4
PtAV-13394	85.5<br>86.7	89.5<br>90.2		99.9<br>99.9	89.4<br>90.4	89.5<br>90.4	89.4<br>90.6	92.7<br>93.3	89.2<br>90.4	41.2<br>47.4	89.3<br>90.1
PtAV-13395-1	85.8<br>87.2	89.5<br>90.3	99.2<br>99.3		89.4<br>90.4	89.5<br>90.4	89.5<br>90.8	92.8<br>93.5	89.3<br>90.5	41.2<br>47.4	89.4<br>90.2
PtAV-13395-2	94.6<br>94.8	84.9<br>86.5	85.9<br>87.0	86.1<br>87.4		99.7<br>99.7	96.8<br>96.8	88.9<br>90.5	96.8<br>96.8	40.8<br>46.9	99.3<br>99.2
PtAV-13396	94.7<br>94.9	85.0<br>86.5	86.0<br>87.0	86.1<br>87.2	99.6<br>99.7		97.1<br>97.0	88.9<br>90.5	97.1<br>97.0	40.8<br>46.9	99.6<br>99.5
PtAV-13421	99.1<br>99.0	85.1<br>86.5	85.3<br>86.5	85.6<br>87.0	94.8<br>95.0	95.0<br>95.0		89.0<br>90.8	99.6<br>99.5	40.8<br>46.9	97.0<br>96.9
PtAV-13430	85.1<br>86.4	99.4<br>99.3	89.5<br>90.2	89.5<br>90.2	85.0<br>86.5	85.0<br>86.4	85.0<br>86.4		88.9<br>90.5	41.0<br>46.9	88.9<br>90.4
PtAV-13438-1	99.3<br>99.2	84.9<br>86.4	85.3<br>86.6	85.7<br>87.1	94.7<br>95.0	94.8<br>95.0	99.3<br>99.4	84.9<br>86.3		40.7<br>46.8	97.2<br>97.2
PtAV-13438-2	48.2<br>50.8	49.3<br>52.2	48.7<br>51.9	48.8<br>52.1	47.8<br>50.6	47.8<br>50.7	48.2<br>50.9	49.3<br>52.2	48.1<br>50.9		40.7<br>46.8
PtAV-13442	94.8<br>95.0	85.0<br>86.5	86.2<br>87.2	86.4<br>87.5	98.7<br>99.0	98.9<br>99.1	95.0<br>95.1	85.0<br>86.5	94.9<br>95.2	47.9<br>50.6	

Nucleic acid identities (in black font) of the coding region (the genome sequence excluding the 5' and 3' untranslated regions) and ORF2 are shown in the bottom left half of the table while amino acid identities (in red font) of the predicted ORF1–ORF2 fusion protein and the ORF2 product are shown in the top right half. In each cell, the value on the top is the pairwise identity of the coding region/fusion protein while the one at the bottom is the pairwise identity ORF2 or its product.

Table S13. Closest matches of the predicted Pterostylis amalgavirus fusion protein and ORF2 product from BLASTP searches of the NCBI non-redundant protein sequence database.

Virus Protein	Isolate	Virus Name of the Closest Hit	GenBank Accession Number of the Closest Hit	E-value	Query Coverage (%)	Pairwise Identity (%)
ORF1–ORF2 Fusion Protein	PtAV-13365	Festuca pratensis amalgavirus 3	DAB41731.1	0.0	95	45.7
	PtAV-13392	Festuca pratensis amalgavirus 3	DAB41731.1	0.0	95	46.4
	PtAV-13394	Camellia totivirus A *	QED42799.1	0.0	96	47.9
	PtAV-13395-1	Camellia totivirus A *	QED42799.1	0.0	96	47.9
	PtAV-13395-2	Festuca pratensis amalgavirus 3	DAB41731.1	0.0	94	46.1
	PtAV-13396	Festuca pratensis amalgavirus 3	DAB41731.1	0.0	95	45.8
	PtAV-13421	Festuca pratensis amalgavirus 3	DAB41731.1	0.0	95	46.0
	PtAV-13430	Festuca pratensis amalgavirus 3	DAB41731.1	0.0	95	46.3
	PtAV-13438-1	Festuca pratensis amalgavirus 3	DAB41731.1	0.0	95	45.8
	PtAV-13438-2	Gevuina avellana amalgavirus 1	DAB41733.1	0.0	99	53.5
	PtAV-13442	Festuca pratensis amalgavirus 3	DAB41731.1	0.0	95	45.7
Product of ORF2	PtAV-13365	Zostera marina amalgavirus 2	YP_009362304.1	0.0	92	56.6
	PtAV-13392	Camellia totivirus A *	QED42799.1	0.0	98	53.7
	PtAV-13394	Camellia totivirus A *	QED42799.1	0.0	95	54.7
	PtAV-13395-1	Camellia totivirus A *	QED42799.1	0.0	95	54.7
	PtAV-13395-2	Zostera marina amalgavirus 2	YP_009362304.1	0.0	93	56.7
	PtAV-13396	Zostera marina amalgavirus 2	YP_009362304.1	0.0	93	56.7
	PtAV-13421	Zostera marina amalgavirus 2	YP_009362304.1	0.0	92	56.7
	PtAV-13430	Camellia totivirus A *	QED42799.1	0.0	98	53.7
	PtAV-13438-1	Zostera marina amalgavirus 2	YP_009362304.1	0.0	92	56.7
	PtAV-13438-2	Gevuina avellana amalgavirus 1	DAB41733.1	0.0	99	59.0
	PtAV-13442	Zostera marina amalgavirus 2	YP_009362304.1	0.0	93	56.5

* Homology search suggested the virus should be classified as an amalgavirus rather than a totivirus

Table S14. Pairwise sequence identities (%) of the totiviruses identified in this study.

	Pt-TV-13365	Pt-TV-13421-1	Pt-TV-13421-2	Pt-TV-GF	Pt-TV-HR
Pt-TV-13365		95.0	66.4	94.9	94.9
Pt-TV-13421-1	80.6		66.4	97.7	97.7
Pt-TV-13421-2	64.4	64.4		66.5	66.3
Pt-TV-GF	80.8	86.3	64.1		99.4
Pt-TV-HR	80.4	86.2	63.9	98.0	

Nucleic acid identities (in black font) of the coding regions (the genome excluding the 5' and 3' untranslated regions) are shown in the bottom left half of the table while amino acid identities (in red font) of the ORF1–ORF2 fusion protein are shown in the top right half.

Table S15. Closest matches of the predicted Pterostylis totivirus fusion protein and ORF2 product from BLASTP searches of the NCBI non-redundant protein sequence database.

Virus Protein	Isolate	Virus Name of the Closest Hit	GenBank Accession Number of the Closest Hit	E-value	Query Coverage (%)	Pairwise Identity (%)
ORF1–ORF2 Fusion Protein	PtTV-13365	maize-associated totivirus 3	AUH27273.1	0.0	99	43.5
	PtTV-13421-1	maize-associated totivirus 3	AUH27273.1	0.0	99	43.8
	PtTV-13421-2	maize-associated totivirus 3	AUH27273.1	0.0	99	43.5
	PtTV-GF	maize-associated totivirus 3	AUH27273.1	0.0	99	43.5
	PtTV-HR	maize-associated totivirus 3	AUH27273.1	0.0	99	43.5
Product of ORF2	PtTV-13365	Pterostylis sanguinea totivirus A	AOX47551.1	0.0	91	69.9
	PtTV-13421-1	Pterostylis sanguinea totivirus A	AOX47551.1	0.0	91	69.8
	PtTV-13421-2	Pterostylis sanguinea totivirus A	AOX47551.1	0.0	91	68.6
	PtTV-GF	Pterostylis sanguinea totivirus A	AOX47551.1	0.0	91	69.7
	PtTV-HR	Pterostylis sanguinea totivirus A	AOX47551.1	0.0	91	69.7

Table S16. Closest matches of the predicted Pterostylis polerovirus proteins from BLASTP searches of the NCBI non-redundant protein sequence database.

Virus Protein	Virus Name of the Closest Hit	GenBank Accession Number of the Closest Hit	E-value	Query Coverage (%)	Pairwise Identity (%)
P0	cereal yellow dwarf virus RPS	QGT40983.1	3E-08	78	29.4
P1	tobacco yellow virus	QIZ03428.1	4E-126	99	38.4
P2 *	potato leafroll virus	AAL77939.1	0.0	89	67.2
P1–P2	potato leafroll virus	ARS33717.1	0.0	99	51.1
P3a	beet mild yellowing virus	QYA72330.1	4E-14	97	68.9
P3	cereal yellow dwarf virus RPV	QDM54317.1	5E-78	99	68.1
P4	potato leafroll virus	ABY49849.1	6E-39	98	64.6
P3–P5	Brassica yellows virus	BBG75785.1	0.0	90	55.4

* Product of ORF2

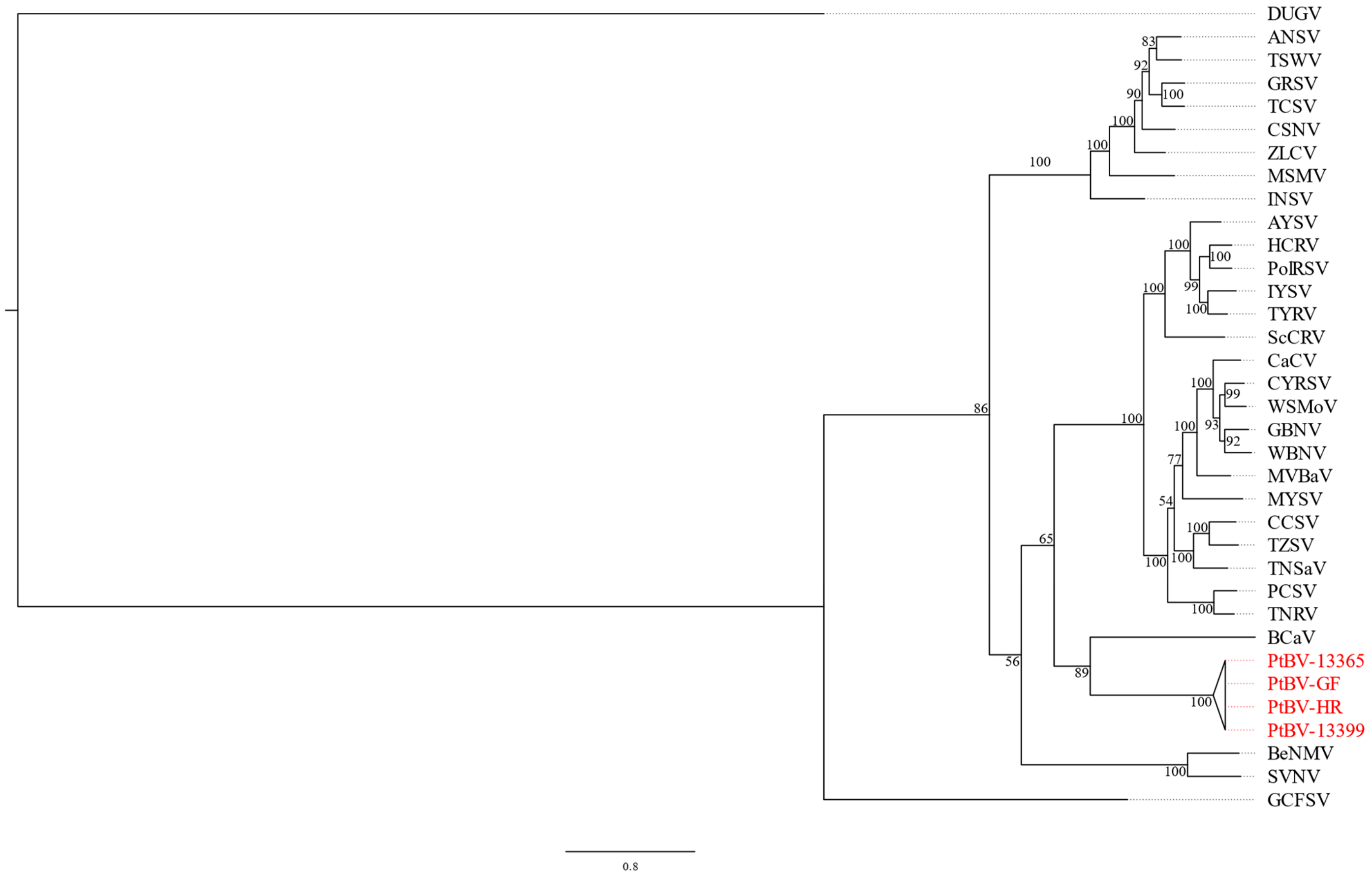

Figure S1. Maximum likelihood phylogenetic tree based on RNA dependent RNA polymerase gene rooted to Dugbe virus (DUGV). The nucleotide substitution model used was GTR+F+R4. The acronyms in red font correspond to Pterostylis blotch virus (PtBV) sequences. The other virus acronyms are defined in Table S3. The numbers next to the branches are non-parametric bootstrap values based on 1000 replicates.

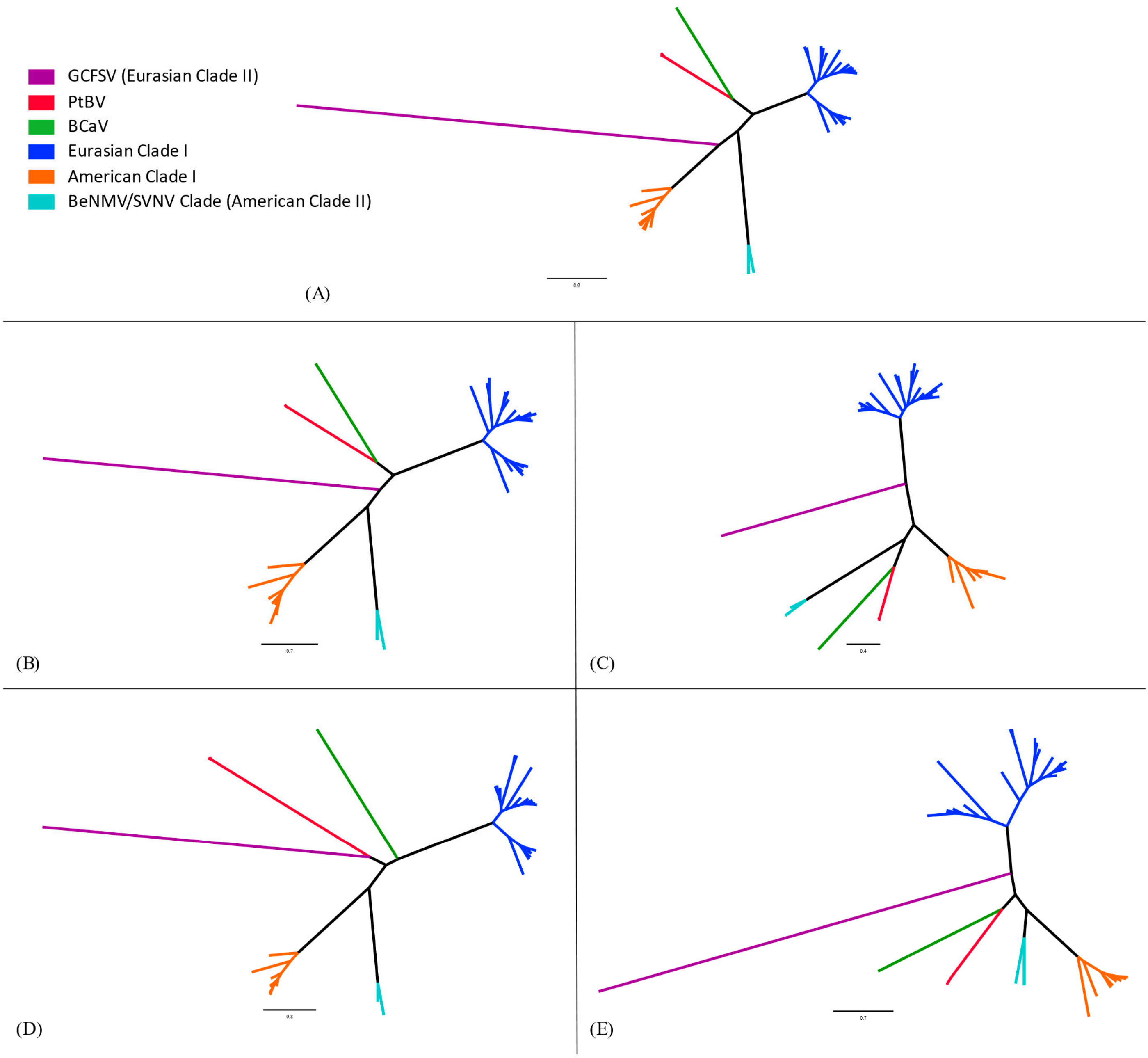

Figure S2. Maximum likelihood phylogenetic gene trees of orthotospoviruses. (A) RNA dependent RNA polymerase; substitution model: GTR+F+R6. (B) Non-structural protein, M segment; substitution model: GTR+F+R5. (C) Glycoprotein precursor; substitution model: GTR+F+G4. (D) Non-structural protein, S segment; substitution model: TPM3+F+I+G4. (E) Nucleocapsid protein; substitution model: GTR+F+I+G4. GCFSV, groundnut chlorotic fan-spot virus; PtBV, Pterostylis blotch virus; BCaV, Barleria chlorosis-associated virus; BeNMV, bean necrotic mosaic virus; SVNV, soybean vein necrosis virus.

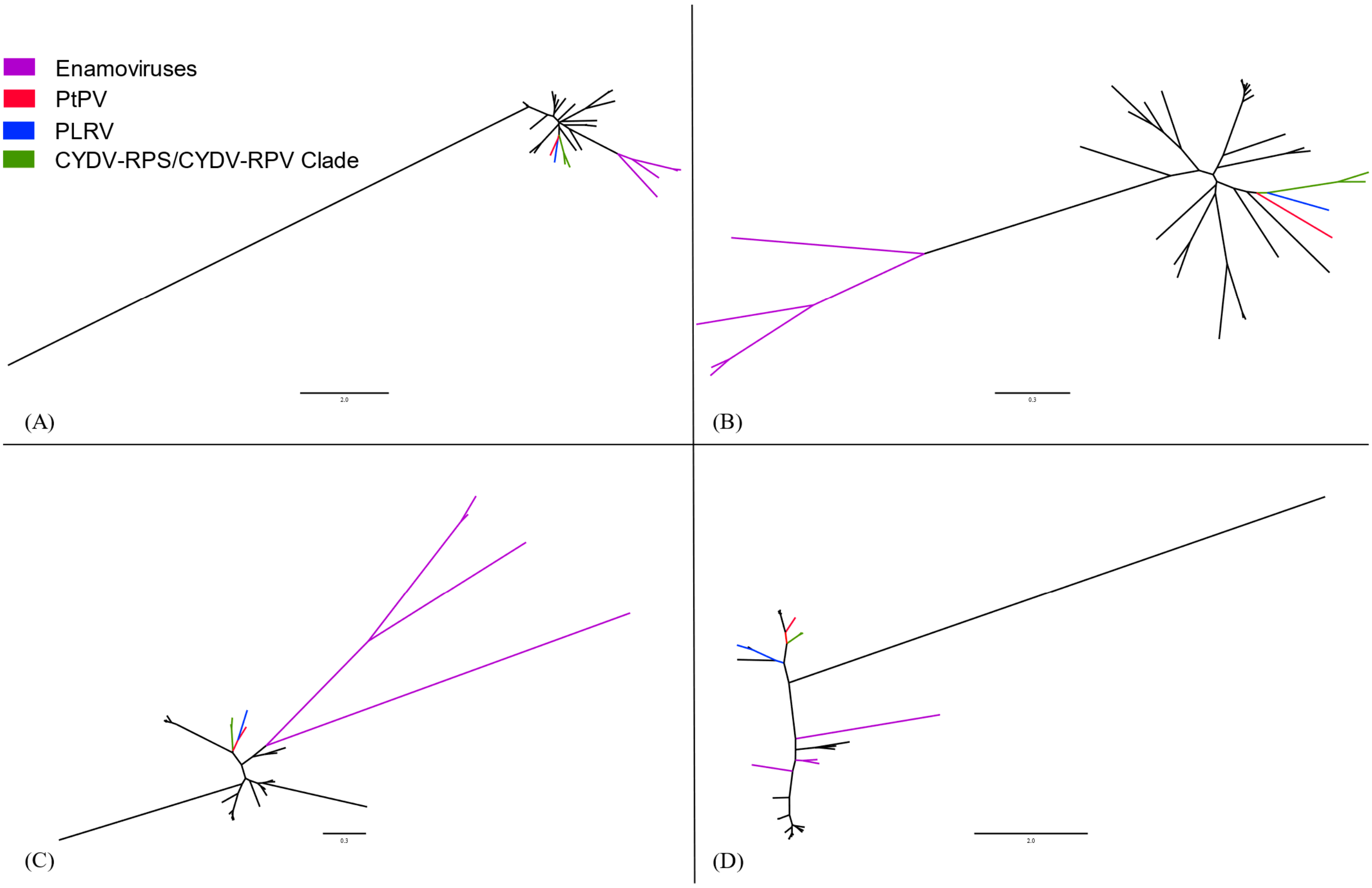

Figure S3. Maximum likelihood phylogenetic gene trees of poleroviruses and enamoviruses. (A) Conserved domain of serine proteinase; substitution model: TIM2e+R4. (B) Conserved domain of RNA dependent RNA polymerase; substitution model: SYM+I+G4. (C) Conserved domain of major capsid protein; substitution model: TIM2e+G4. (D) Conserved domain of minor capsid protein; substitution model: TIM2e+G4. PtPV, Pterostylis polerovirus; PLRV, potato leafroll virus; CYDV, cereal yellow dwarf virus.
